# Gastrointestinal adverse events during methylphenidate treatment of children and adolescents with attention deficit hyperactivity disorder: A systematic review with meta-analysis and Trial Sequential Analysis of randomised clinical trials

**DOI:** 10.1371/journal.pone.0178187

**Published:** 2017-06-15

**Authors:** Mathilde Holmskov, Ole Jakob Storebø, Carlos R. Moreira-Maia, Erica Ramstad, Frederik Løgstrup Magnusson, Helle B. Krogh, Camilla Groth, Donna Gillies, Morris Zwi, Maria Skoog, Christian Gluud, Erik Simonsen

**Affiliations:** 1 Psychiatric Research Unit, Region Zealand Psychiatry, Slagelse, Denmark; 2 Child and Adolescent Psychiatric Department, Region Zealand, Denmark; 3 Psychological Institute, Faculty of Health Science, University of Southern Denmark, Odense, Denmark; 4 Federal University of Rio Grande do Sul, Porto Alegre, Brazil; 5 Pediatric Department E, Herlev University Hospital, Herlev, Denmark; 6 Western Sydney Local Health District; Mental Health, Parramatta, Australia; 7 Islington CAMHS, Whittington Health, London, United Kingdom; 8 Copenhagen Trial Unit, Centre for Clinical Intervention Research, Rigshospitalet, Copenhagen University Hospital, Copenhagen, Denmark; 9 The Cochrane Hepato-Biliary Group, Copenhagen Trial Unit, Centre for Clinical Intervention Research, Department 7812, Rigshospitalet, Copenhagen University Hospital, Copenhagen, Denmark; 10 Institute of Clinical Medicine, Faculty of Health and Medical Sciences, Copenhagen University, Copenhagen, Denmark; Cardiff University, UNITED KINGDOM

## Abstract

**Objectives:**

To study in more depth the relationship between type, dose, or duration of methylphenidate offered to children and adolescents with attention deficit hyperactivity disorder and their risks of gastrointestinal adverse events based on our Cochrane systematic review.

**Methods and findings:**

We use data from our review including 185 randomised clinical trials. Randomised parallel-group trials and cross-over trials reporting gastrointestinal adverse events associated with methylphenidate were included. Data were extracted and quality assessed according to Cochrane guidelines. Data were summarised as risk ratios (RR) with 95% confidence intervals (CI) using the inverse variance method. Bias risks were assessed according to domains. Trial Sequential Analysis (TSA) was used to control random errors.

Eighteen parallel group trials and 43 cross-over trials reported gastrointestinal adverse events. All trials were at high risk of bias. In parallel group trials, methylphenidate decreased appetite (RR 3.66, 95% CI 2.56 to 5.23) and weight (RR 3.89, 95% CI 1.43 to 10.59). In cross-over trials, methylphenidate increased abdominal pain (RR 1.61, 95% CI 1.27 to 2.04). We found no significant differences in the risk according to type, dose, or duration of administration. The required information size was achieved in three out of four outcomes.

**Conclusion:**

Methylphenidate increases the risks of decreased appetite, weight loss, and abdominal pain in children and adolescents with attention deficit hyperactivity disorder. No differences in the risks of gastrointestinal adverse events according to type, dose, or duration of administration were found.

## Introduction

Attention deficit hyperactivity disorder (ADHD) is one of the most common neuro-developmental disorders in children and adolescents with an estimated worldwide prevalence of 5.3% to 7.2% [1,2]. Methylphenidate is widely used as the first line drug in the treatment of ADHD in children and adolescents [3–5].

Gastrointestinal adverse events are well-described in association with methylphenidate administration to children and adolescents with ADHD. The most frequently reported adverse events encompass decreased appetite, abdominal pain, diarrhoea, and nausea [3,6,7]. Earlier studies differ regarding the type of methylphenidate preparation and risks of gastrointestinal adverse events. Some studies report no difference in gastrointestinal adverse events, specifically decreased appetite, between immediate release and extended release formulations of methylphenidate [8–11]. Other studies suggest switching to another stimulant medication if the child experience adverse events [7,12]. The consensus though seems to be an agreement to lower or adjust the dose of methylphenidate if a patient experience gastrointestinal adverse events. Wolraich et al. suggested to decrease the dose of methylphenidate if the child experience abdominal pain [7]. Pliszka et al. suggested dose adjustment of the stimulant as a strategy for dealing with adverse events in methylphenidate treatment [12].

As we could not find a clear relationship between the risk of gastrointestinal adverse events and preparation type, duration of treatment, or methylphenidate dose in the literature, we based the current study on our recent Cochrane systematic review on methylphenidate for children and adolescents with ADHD [13–15]. The published Cochrane systematic review does not include subgroup analyses regarding the risks of gastrointestinal adverse events according to type, dose, and duration of methylphenidate administration. Our review only includes analyses regarding the risk of gastrointestinal adverse events overall. Therefore, we found it worthwhile to analyse the gastrointestinal adverse events in more detail.

## Methods

This paper presents additional data and analyses focusing on gastrointestinal adverse events in our Cochrane systematic review [13–15]. The protocol was published in the Cochrane Library [16].

### Data sources and search criteria

The literature search was carried out in: Cochrane Central Register of Controlled Trials, MEDLINE, EMBASE, CINAHL, PsycINFO, ISI Conference Proceedings Citation Index, Clinical Trials.gov, and International Clinical Trials Registry Platform (ICTRP) from origin and updated up to February 2015.

Furthermore, we screened the reference lists of identified review articles, meta-analyses, and a sample of included trials. Requests for published as well as unpublished data were sent to pharmaceutical companies manufacturing methylphenidate. We also requested unpublished studies from authors.

Further details on the data sources and search criteria are available at the Cochrane systematic review [13–15].

### Study selection

We included randomised clinical trials describing children and adolescents diagnosed with ADHD according to the Diagnostic and Statistical Manual of Mental Disorders [17–21] or with hyperkinetic disorders according to the International Classification of Disease [22,23]. At least 75% of the study participants had to be younger than 19 years and the mean age had to be below 19 years. We included trials irrespective of comorbidities but at least 75% of the participants were required to have an intellectual capacity (IQ) above 70 points.

Trials assessing any methylphenidate presentation were included (immediate or extended release, and transdermal system), and as comparator both placebo and no intervention were accepted. Co-interventions were permitted if both groups received them and there were no limitations on language, publication year, publication type, or publication status. Screening and inclusion of studies were carried out according to *The Cochrane Handbook for Systematic Reviews of Interventions* [13,24]. We made sure that data on patients from articles based on the same randomised trial were only used once.

In the present paper, we selected the randomised clinical trials reporting on gastrointestinal adverse events, e.g., decreased or increased appetite, abdominal pain, diarrhoea, nausea, vomiting, dry mouth, dyspepsia, and weight loss. The adverse events were measured by using adverse events rating scales, e.g., Barkley Side Effects Rating Scale [25], spontaneous reports, and/or recorded by the investigators at regular interviews.

### Data extraction and quality assessment

Data were extracted from all included trials, which reported gastrointestinal adverse events. Seventeen review authors extracted data, working together in groups of two independent data extractors. Disagreements were resolved by discussion and we used an arbiter if required. Before we embarked on data extraction, we developed data extraction forms (after performing data extraction pilots, we updated these forms to accommodate extraction of more detailed data and to facilitate standardised approaches to data extraction among review authors). All data extractors used these extraction forms [13]. Risk of bias of the individual trials was assessed according to *The Cochrane Handbook for Systematic Reviews of Interventions* [24]. For each included trial, data extractors independently evaluated risk of bias domains, resolving disagreements by discussion. We assessed and graded the quality of the evidence according to the Grading of Recommendations, Assessment, Development, and Evaluation (GRADE) [13,26].

### Data synthesis and statistical analysis

Data from the parallel group trials were dichotomous and summarized as risk ratio (RR) with 95% confidence interval (CI), using the inverse variance method. Data from cross-over trials were both dichotomous and continuous and were combined using the generic inverse variance method and reported as RR with 95% CI. We used end of trial data from all cross-over trials. Random-effects model was used. To test for possible carry-over effect and unit of analysis error in the use of end of trial data from cross-over trials we made a subgroup analysis comparing data on decreased appetite from parallel group trials with end of trial data from cross-over trials. To test for possible effect of co-medication on the risk of gastrointestinal adverse events we conducted subgroup analyses comparing trials with co-medication to trials without co-medication.

Additional subgroup analyses were executed for type (defined as immediate release or extended release (including modified-release, depot tablets, and transdermal preparations of methylphenidate)), dose (defined as low ≤ 20 mg/day (0.6 mg/kg/day) and moderate/high (> 20mg/day) based on recommended treatment doses of methylphenidate [3,6]). Duration of administration was defined as short-term ≤ 8 weeks (56 days) and long-term > 8 weeks according to the NICE guidelines [3].

Publication bias was assessed by testing for funnel plot asymmetry and performing Eggers’ statistical test for outcomes including at least 10 parallel group trials in the meta-analysis [24].

A meta-analysis should include a calculation of a required information size at least as large as the sample size of an adequately powered single trial to reduce the risks of random errors, taking into consideration the inconsistency of the meta-analysis [27–29]. Trial Sequential Analysis (TSA) is a program that calculates the required information size for a meta-analysis, providing adjusted statistical thresholds for benefits, harms, or futility before the required information size is reached. TSA can thereby control the risks of type I and type II errors due to sparse data and repetitive testing of accumulating data [27,28,30–32]. We used TSA to estimate the required information size for the outcomes: decreased appetite, abdominal pain, nausea, and vomiting.

The data were analysed using Review Manager [33].

## Results

### Trial selection

**Fig 1:**
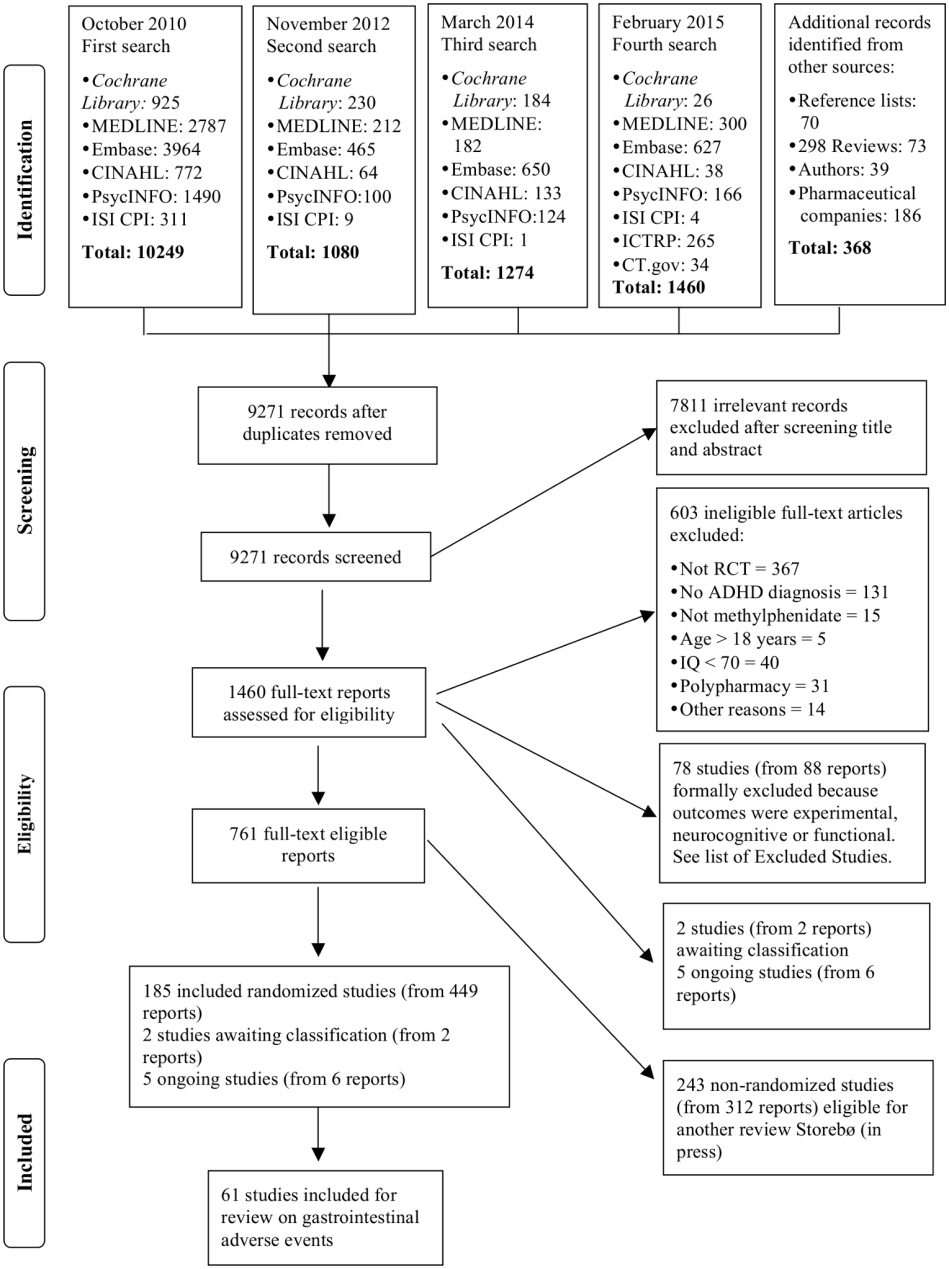
PRISMA flow chart [34].

A total of 61 randomised trials (33%), 18 parallel group trials and 43 cross-over trials, described gastrointestinal adverse events out of the 185 trials included in our review [13–15]. The PRISMA flow chart (Fig 1 [34]) shows the trials identified, screened, and included in this systematic review.

### Description of included trials

The description of the included trials can be seen in Table 1. Further characteristics of the included parallel group trials and cross-over trials and full list of included trials are shown in the supporting information (S1 and S2 Tables; S1 Text).

**Table 1:** Description of the included trials on methylphenidate for children and adolescents with attention deficit hyperactivity disorder.

	Parallel group trials	Cross-over trials
Number of studies	18	43
Number of publications	57	113
Number of participants	3,564	2,419
Mean age (range)	10.4 (6 to 18) years	9.4 (3 to 18) years
Number of males (%)	2,643 (74.2)	1,747 (72.2)
Type of methylphenidate*	IR-MPH: 3 studies ER-MPH: 13 studies IR- and ER-MPH: 2 studies	IR-MPH: 15 studies ER-MPH: 14 studies IR- and ER-MPH: 6 studies Unknown: 8 studies
Dose range of methylphenidate (mg/day)	10 to 68	5 to 75
Number of trials reporting on low and high dose**	Low dose: 0 trials High dose: 8 trials Both low and high dose: 3 trials Unknown dose: 7 trials	Low dose: 16 trials High dose: 6 trials Both low and high dose: 17 trials Unknown dose: 4 trials
Mean duration of treatment (days)	43	15.6
Number of trials of short-term and long-term duration***	Short term: 15 trials Long term: 2 trials Unknown duration: 1 trial	Short term: 43 trials Long term: 0 trials

* ER-MPH: extended-release methylphenidate (including modified-release, depot tablets, and transdermal preparations of methylphenidate); IR-MPH: immediate-release methylphenidate.

** Low dose: ≤ 20 mg/day (0.6 mg/kg/day); high dose: > 20 mg/day.

*** Short-term duration ≤ 8 weeks (56 days); long-term duration > 8 weeks.

### Risk of bias assessment of included trials

All 61 trials included were at high risk of bias, according to *The Cochrane Handbook for Systematic Reviews of Interventions* [24]. Three cross-over trials had low risk of bias in all domains [35–37], but as the blinding may have been easily broken in these trials owing to prevalent adverse events, we also consider these trials at high risk of bias [13–15].

### Gastrointestinal adverse effects of methylphenidate compared with placebo or no intervention in parallel group trials

**Fig 2:**
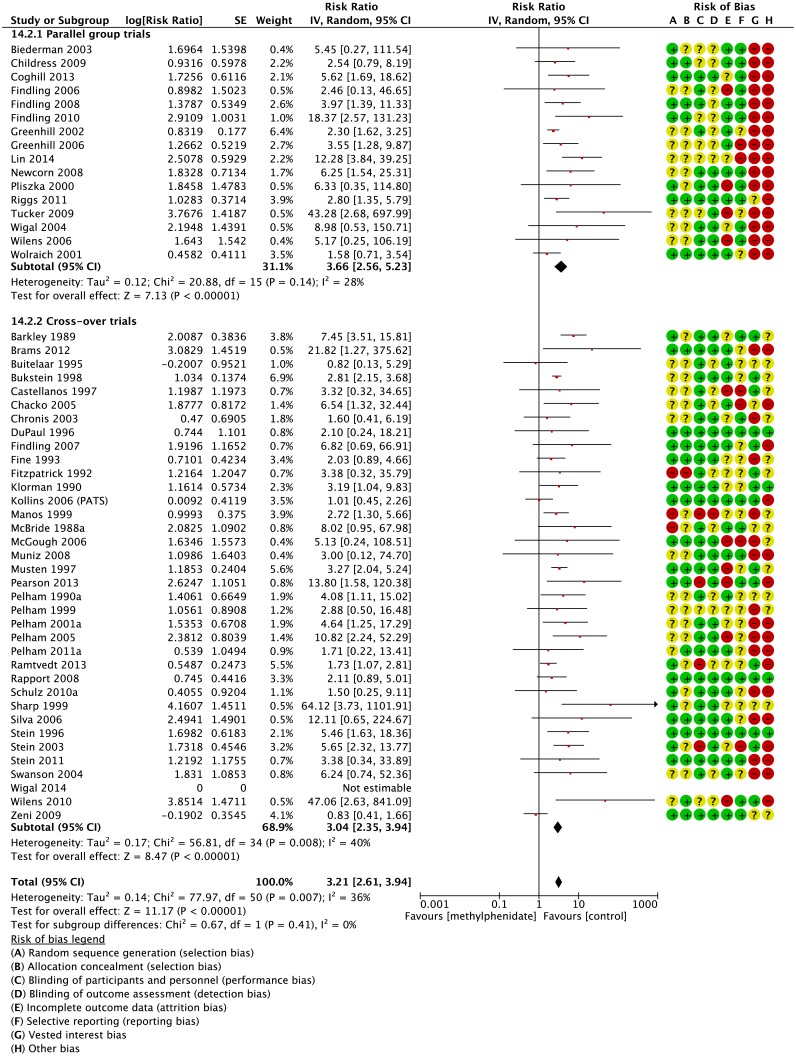
Parallel group trials and cross-over trials: Risk of decreased appetite. IV: inverse variance. Random: random-effect model. CI: confidence interval. The risk of bias items was rated as low (plus), unclear (question mark) or high risk of bias (minus): A: Random sequence generation (selection bias). B: Allocation concealment (selection bias). C: Blinding of participants and personnel (performance bias). D: Blinding of outcome assessment (detection bias). E: Incomplete outcome data (attrition bias). F: Selective reporting (reporting bias). G: Vested interest.

**Fig 3:**
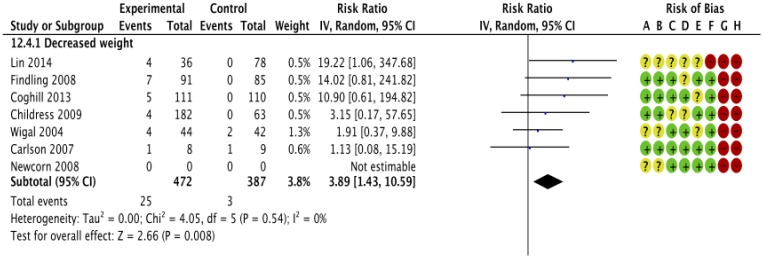
Parallel group trials: Risk of decreased weight. IV: inverse variance, Random: random-effect model. CI: confidence interval. The risk of bias items was rated as low (plus), unclear (question mark) or high risk of bias (minus): A: Random sequence generation (selection bias). B: Allocation concealment (selection bias). C: Blinding of participants and personnel (performance bias). D: Blinding of outcome assessment (detection bias). E: Incomplete outcome data (attrition bias). F: Selective reporting (reporting bias). G: Vested interest.

In parallel group trials methylphenidate was associated with decreased appetite (RR 3.66; 95% CI 2.56 to 5.23; 2,962 participants; 16 trials, Fig 2) and decreased weight (RR 3.89; 95% CI 1.43 to 10.59; 859 participants; 7 trials, Fig 3). Participants did not experience increased or decreased risk of any of the following gastrointestinal adverse events: diarrhoea (RR 1.07; 95% CI 0.41 to 2.77; 5 trials), dyspepsia (RR 1.80; 95% CI 0.71 to 4.54; 2 trials), increased appetite (RR 0.07; 95% CI 0.00 to 1.43; 1 trial), nausea (RR 1.30; 95% CI 0.85 to 1.99; 11 trials), abdominal pain (RR 1.30; 95% CI 1.00 to 1.69; 13 trials), and vomiting (RR 1.17; 95% CI 0.76 to 1.79; 11 trials) (Graphics are shown in the supporting information (S1 Figs)).

We found no difference in the risk of any gastrointestinal adverse events according to type of methylphenidate preparation: decreased appetite (P=0.11), decreased weight (P=0.28), dyspepsia (P=0.98), nausea (P=0.20), abdominal pain (P=0.72), and vomiting (P=0.85) (Graphics are shown in the supporting information (S2 Figs)). We were not able to make a subgroup analysis on diarrhoea as all participants in this group received extended release methylphenidate.

**Fig 4:**
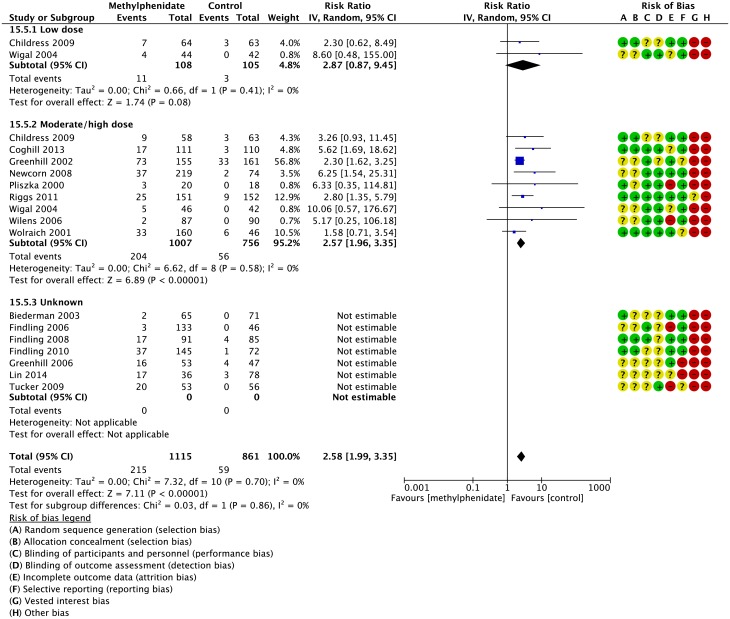
Parallel group trials: Risk of decreased appetite according to dose of methylphenidate. IV: inverse variance, Random: random-effects model. CI: confidence interval. The risk of bias items was rated as low (plus), unclear (question mark) or high risk of bias (minus): A: Random sequence generation (selection bias). B: Allocation concealment (selection bias). C: Blinding of participants and personnel (performance bias). D: Blinding of outcome assessment (detection bias). E: Incomplete outcome data (attrition bias). F: Selective reporting (reporting bias). G: Vested interest. Low dose: ≤ 20 mg/day (0.6 mg/kg/day); high dose: > 20 mg/day (0.6 mg/kg/day).

We found no significant difference in the risk of decreased appetite between trials using low dose (RR 2.87; 95% CI 0.87 to 9.45) and moderate/high dose (RR 2.57; 95% CI 1.96 to 3.35), (test for subgroup differences: P=0.86), with an increased risk of decreased appetite only in the moderate/high dose group. Seven studies did not report the dose of methylphenidate used (Fig 4).

We found no significant difference in the risk of any other gastrointestinal adverse events according to dose: decreased weight (P=0.72), diarrhea (P=0.94), nausea (P=0.63), abdominal pain (P=0.95), and vomiting (P=0.68) (Graphics are shown in the supporting information (S3 Figs)). Furthermore, we were not able to do the subgroup analysis on dyspepsia since we had no information on dose of methylphenidate in any of the studies reporting on this outcome.

We found no significant difference in the risk of any gastrointestinal adverse events when comparing short-term with long-term trials: decreased appetite (P=0.53), dyspepsia (P=0.98), nausea (P=0.38), abdominal pain (P=0.18), and vomiting (P=0.95) (Graphics are shown in the supporting information (S4 Figs)). We were not able to do the subgroup analysis on decreased weight and diarrhoea as all the trials reporting this outcome were of short-term duration.

### Gastrointestinal adverse effects of methylphenidate compared with placebo or no intervention in cross-over trials

**Fig 5:**
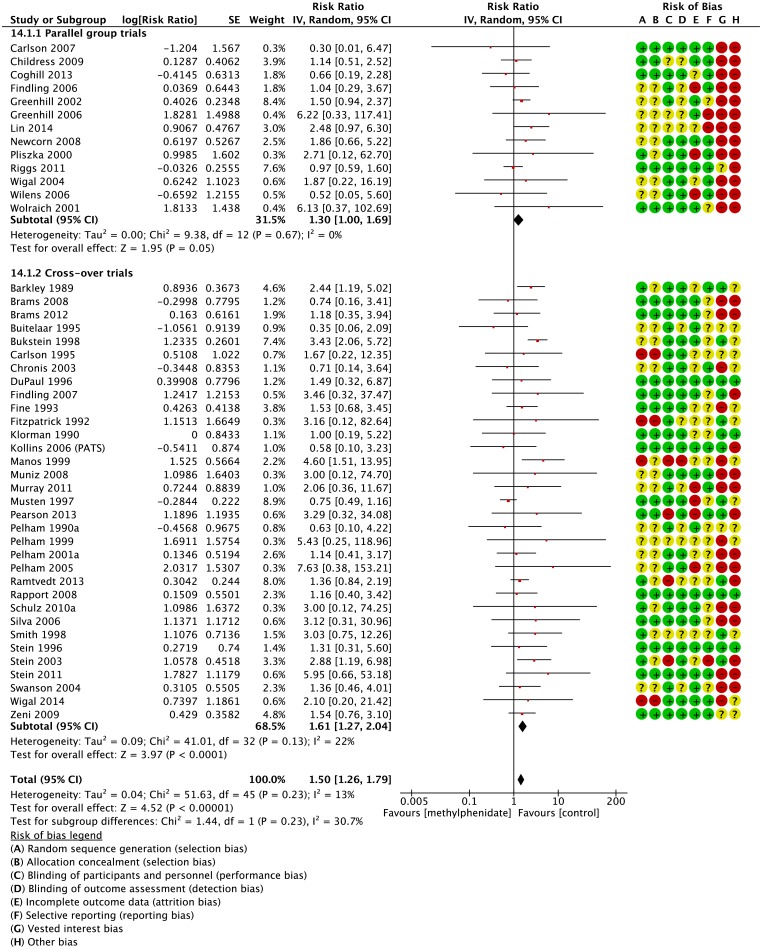
Parallel group trials and cross-over trials: Risk of abdominal pain. IV: inverse variance, Random: random-effects model. CI: confidence interval. The risk of bias items was rated as low (plus), unclear (question mark) or high risk of bias (minus): A: Random sequence generation (selection bias). B: Allocation concealment (selection bias). C: Blinding of participants and personnel (performance bias). D: Blinding of outcome assessment (detection bias). E: Incomplete outcome data (attrition bias). F: Selective reporting (reporting bias). G: Vested interest.

We found that methylphenidate decreased appetite (RR 3.04; 95% CI 2.35 to 3.94; 1,901 participants; 36 trials, Fig 2) and increased abdominal pain (RR 1.61; 95% CI 1.27 to 2.04; 1,837 participants; 33 trials, Fig 5). One trial observed that methylphenidate increased appetite (RR 0.20; 95% CI 0.08 to 0.50; 68 participants; 1 trial [11]). Participants did not experience an increased or decreased risk of any other gastrointestinal adverse events (Graphics are shown in supporting information (S1 Figs)).

We found no significant difference when comparing the risk of various gastrointestinal adverse events according to type or dose of methylphenidate.

We were not able to perform subgroup analyses on duration of methylphenidate treatment as all cross-over trials were short-term trials (mean = 15.6 days).

### Parallel-group trials compared to cross-over trials

We found no significant difference in the risk of the main outcome decreased appetite between parallel-group and cross-over trials (P = 0.41; I^2^ = 0%; Fig 2). Furthermore, we found no difference in the risk of abdominal pain between parallel group and cross-over trials (P=0.23; Fig 5).

### Possible effect of co-interventions on the risk of gastrointestinal adverse events in parallel group trials and cross-over trials

Only two parallel group trials [38,39] and two cross-over trials [40,41] used co-interventions.

We conducted subgroup analyses comparing these trials to all trials not including co-interventions. None of these tests showed significant subgroup difference when comparing a subgroup including trials with co-interventions to a subgroup excluding these trials (Graphics are shown in supporting information (S5 Figs).

### Publication bias

**Fig 6:**
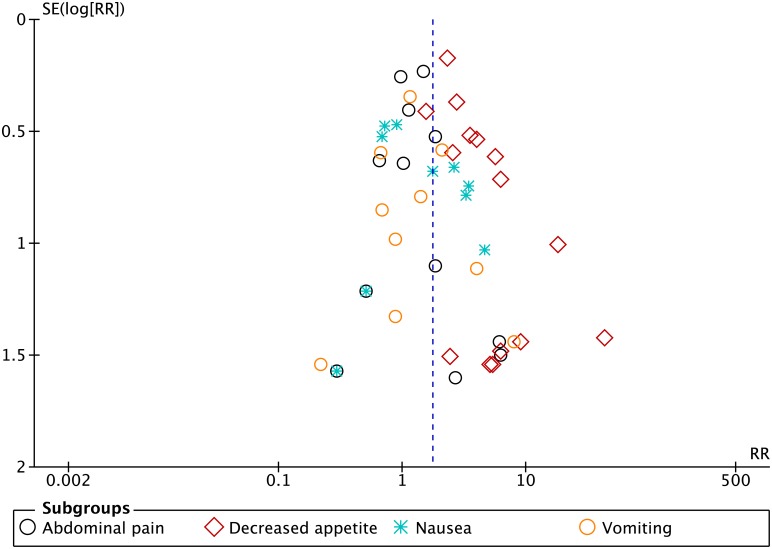
Funnel plot, parallel group trials: Decreased appetite, nausea, abdominal pain, and vomiting. SE: standard error; log: logarithm; RR: relative risk.

Asymmetry of the funnel plots for the outcomes including at least 10 parallel group trials (Fig 6) and the Eggers’ test suggested publication bias for decreased appetite: Eggers’ regression intercept (bias) was 8.717 (two-tailed P = 0.020). Eggers’ test for the outcomes nausea (Egger’s regression intercept (bias) was 2.16 (two-tailed P = 0.215)), abdominal pain (Egger’s regression intercept (bias) was 1.11 (two-tailed P = 0.136)), and vomiting (Egger’s regression intercept (bias) was 1.438 (two-tailed P = 0.271)) showed no statistical significance to conclude whether or not there was publication bias in the meta-analysis on these outcomes (Eggers’ tests are shown in supporting information (S2 Text).

### Trial Sequential Analysis

**Fig 7:**
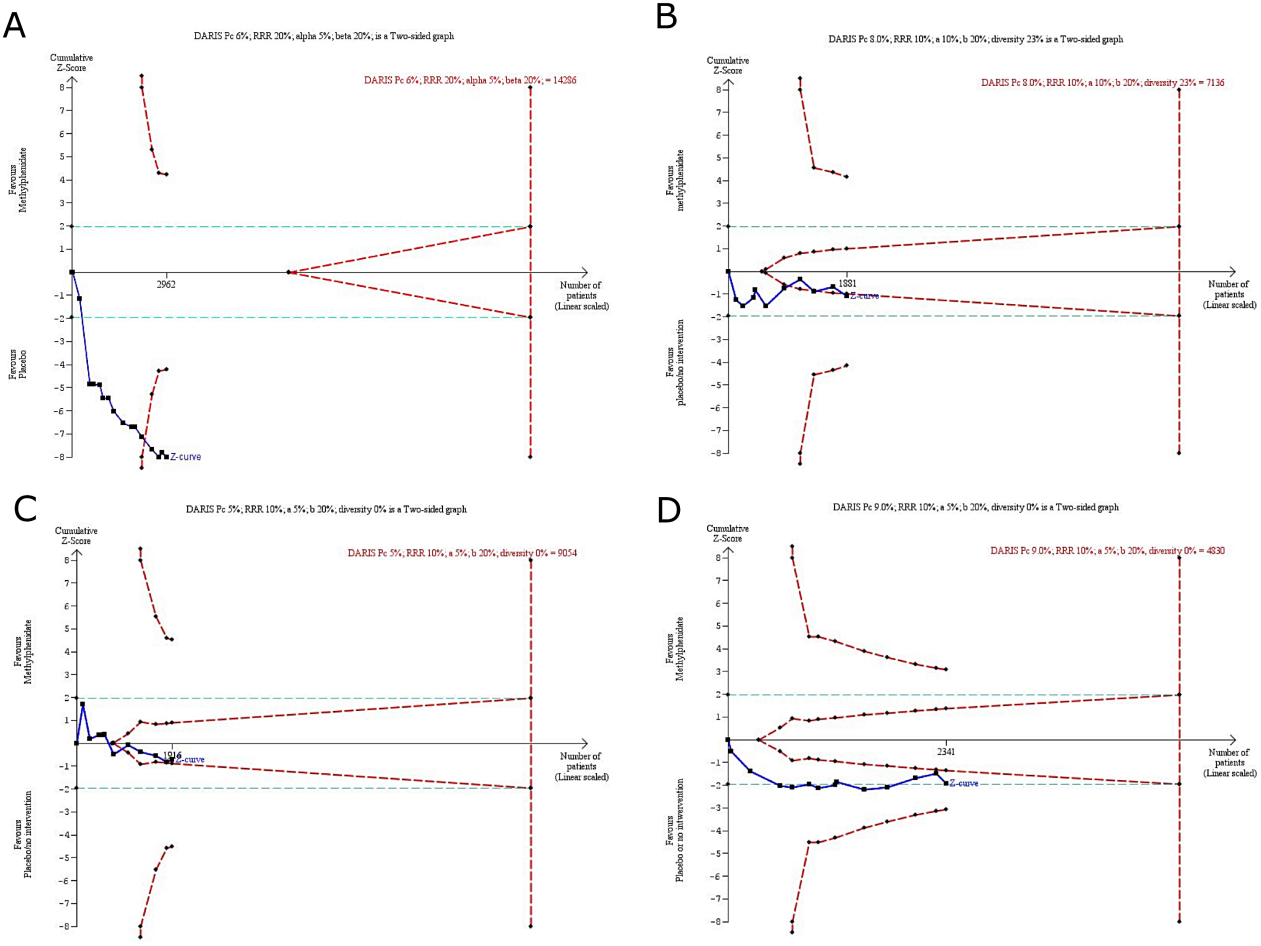
Trial Sequential Analysis, parallel group trials, decreased appetite, nausea, vomiting, and abdominal pain. DARIS: diversity adjusted required information size; Pc: Event proportion in the control group; RRR: relative risk reduction in the intervention group; a: type I error; b: type II error; DIVERSITY: diversity (D-square). A) Decreased appetite: The required information size was 14,286 participants. The cumulative Z-score (dark line) crosses the trial sequential monitoring boundaries for harm (lighter inward sloping line) after the twelfth trial, and stayed below the boundary, thus the risk of random error in the finding can be excluded. Therefore, there may be no need for conducting further trials based on the assumed intervention effect of RRR of 20%, an alpha of 5%, and a beta of 20% regarding this outcome. B) Nausea: The cumulative Z-scores (dark lines) crossed into the areas of futility (in between the two lighter lines). Therefore, there may be no need for conducting further trials based on the assumed intervention effect of RRR of 10%, an alpha of 5%, and a beta of 20%. C) Vomiting: The cumulative Z-scores (dark lines) crossed into the areas of futility (in between the two lighter lines). Therefore, there may be no need for conducting further trials based on the assumed intervention effect of RRR of 10%, an alpha of 5%, and a beta of 20%. D) Abdominal pain: The required information size was 4,830 participants. The cumulative Z curve did not cross the conventional or trial sequential monitoring boundaries for benefit, harm, or futility. Therefore, based on the assumed intervention effect of RRR of 10%, an alpha of 5%, and a beta of 20% we may still need more evidence on this adverse event.

We conducted Trial Sequential Analyses on the outcomes: decreased appetite (16 trials), nausea (11 trials), abdominal pain (13 trials), and vomiting (11 trials) (Fig 7).

For the outcome decreased appetite the required information size was 14,286 participants. The cumulative Z-score (dark line) crosses the trial sequential monitoring boundaries for harm (lighter inward sloping line) after the twelfth trial, and stayed below the boundary, thus the risk of random error in the finding can be excluded (Fig 7A).

For the outcomes nausea and vomiting the cumulative Z-scores (dark lines) crossed into the areas of futility (in between the two lighter lines). Therefore, there was no need for conducting further trials based on the assumed intervention effect of RRR of 10%, an alpha of 5%, and a beta of 20% had the trials been at low risk of bias (Fig 7B and 7C).

For abdominal pain the required information size was 4,830 participants. The cumulative Z curve did not cross the conventional or trial sequential monitoring boundaries for benefit, harm, or futility. Therefore, we still do not know the association between methylphenidate and this adverse event (Fig 7D).

## Discussion

To our knowledge, this is the most comprehensive systematic review and meta-analysis on gastrointestinal adverse events due to methylphenidate use according to type, dose, and duration of the stimulant use in children and adolescents with ADHD in the international literature. Our results showed a risk of decreased appetite, decreased weight, and increased abdominal pain comparing methylphenidate with placebo or no intervention. There was no significant difference in the risk of gastrointestinal adverse events according to type, dose, or duration of methylphenidate administration.

We found no differences in the risk of gastrointestinal adverse events when comparing immediate-release to extended-release methylphenidate, which is in accordance with earlier studies [8–11].

Guidelines on methylphenidate for children and adolescents with ADHD suggest that the risk of decreased appetite may be controlled by dose reduction [6]. Also, Stein et al. found that the proportion of participants who reported severe decreased appetite increased as methylphenidate dose increased from 18 mg/day to 54 mg/day [37]. Our findings suggest that the dose of methylphenidate has no influence on the risk of gastrointestinal adverse events. However, these findings are based on data from less than 10 studies. Moreover, the design of the studies does not allow firm conclusions about the effect of dose reduction on the risk of gastrointestinal adverse events, as we lack randomised clinical trials assessing such benefits.

The subgroup analysis on decreased appetite showed no difference in the estimate and no heterogeneity between parallel-group trials and cross-over trials, suggesting no carry-over effect in the cross-over trials and no unit of analysis error [24].

Both parallel group trials and cross-over trials found an increased risk of decreased appetite, but only the cross-over trials found an increased risk of abdominal pain (RR 1.61; 95% CI 1.27 to 2.04). When testing the statistical difference between these results one will see no statistical subgroup difference: Test for subgroup differences: Chi^2^ = 1.44, df = 1 (P = 0.23), I^2^ = 30.7% (Fig 5). The seemingly contrasting findings between parallel group trials and cross-over trials may in fact not be so much in contrast.

### Limitations and strengths

GRADE assessment on our main comparison decreased appetite in parallel group trials led to downgrading the quality of the evidence to ‘very low quality’ (due to high risk of bias and due to publication bias). This may influence the overall findings of this review, as trials with high risk of bias seem to underestimate harmful effects [42–44]. The funnel plot and Eggers’ tests showed publication bias for the outcome decreased appetite (and no statistical significance to conclude there is publication bias for the outcomes nausea, abdominal pain, and vomiting) meaning that there might be a tendency to underreport decreased appetite in trials examining methylphenidate. These limitations probably mean that this review is underestimating the harmful intervention effects of methylphenidate.

The methods used to assess and evaluate adverse events in the included trials were unsystematic, being a mixture of spontaneous reports and rating scales. The optimal would likely be to use a combination of both.

Our cut-offs for low and moderate/high dose and short-term and long-term duration of administration are somewhat arbitrary. Setting another cut-off might change the results of our subgroup analyses. However, the recommended starting dose of methylphenidate is 10 to 20 mg per day regarding the type of methylphenidate. And the recommended maximum daily maintenance dose of methylphenidate is 54 to 72 mg (or 0.7 mg/kg/dose b.i.d. or t.i.d. [6,12,45]. Also the cut-off of low: < 0.6 mg/kg/day, medium: 0.6-1.0 mg/kg/day, and high: > 1.0 mg/kg/day was previously used in Hazell et al. [46], which is also very similar to Prasad et al. and Schachter et al. [47,48]. The cut-off of < 20mg/day as low and > 20mg medium/high was used in the meta-analysis of Kimko at al. [49] and a similar approach was also used in the well-known systematic review and meta-analysis of King at al. to the Health Technology Assessment (UK) [50]. To sum up, we made all efforts to conduct a systematic review/meta-analysis with similar methods as used in the best studies previously published. We therefore find the cut-off of 0.6 mg/kg/day fair, still recognizing the problems that might be related to this.

In a lot of the trials there was lack of information on the children’s weight. Only two parallel group studies and 11 cross-over trials recorded the dose of methylphenidate in mg/kg. The rest of the studies recorded the dose in mg/day without reporting the weight of the children.

In addition, not all studies provided information on methylphenidate dose and the number of trials reporting on moderate/high dose was three to four times higher.

Furthermore, we are aware of disadvantages associated with dichotomizing continuous outcomes such as a loss of power [51,52], but we did this due to very heterogeneous reporting in the trials.

Many of the cross-over trials also lacked information on type of methylphenidate, likely due to the fact that extended-release methylphenidate was first introduced in 1998. Readers must consider these shortcomings when interpreting the results of this comprehensive meta-analysis.

The trials included in this article are predominantly short-term trials with duration varying from two to six weeks, and only two parallel-group trials of 16 weeks (112 days) were included in the long-term group [38,53]. Consequently, little can be concluded about the risk of gastrointestinal adverse events and decreased weight in methylphenidate treatment longer than these time spans.

Our review also has a number of strengths: first of all, it was conducted as a Cochrane review following the instructions from the Cochrane Handbook [24] and the PRISMA guidelines [34]. Second, a detailed protocol was published prior to the review being conducted [16]. Third, the literature search was comprehensive and systematic. Pharmaceutical companies and the researchers in the field were contacted in order to obtain data from unpublished trials. As result, we think our approach has led to the best possible gathering of relevant literature on the topic. Furthermore, our meta-analysis of parallel group trials showed enough power in three out of four outcomes according to the Trial Sequential Analysis.

## Conclusions

Short-term methylphenidate treatment may increase the risk of decreased appetite, weight loss, and abdominal pain in children and adolescents with ADHD. We have limited knowledge on the risks of gastrointestinal adverse events follow long-term use. There appears to be no difference in the risk of gastrointestinal adverse events, including decreased appetite, due to type, dose or duration of methylphenidate treatment. However, these findings should be interpreted in the light of several limitations including: low quality of evidence for the outcomes (which might underestimate the actual risk of gastrointestinal adverse events); lack of information on type and dose of methylphenidate in the included studies; lack of information on gastrointestinal adverse events from long-term studies. Therefore, even though methylphenidate might be the most-studied psychoactive medication for a specific child mental disorder in the field, further randomised clinical trials of high quality with long study duration, dose and type of methylphenidate clearly reported, and a systematic measuring of gastrointestinal adverse events including weight and height are needed.

## Clinical significance

Methylphenidate seems to increase the risk of decreased appetite, weight, and abdominal pain in children and adolescents with ADHD. All types, doses, and durations of methylphenidate seem to influence the risk of gastrointestinal adverse events. The evidence is limited by serious risk of bias in the included trials and a lack of studies of longer duration.

## Supporting information

S1 Table Characteristics of included parallel group trials. (XLSX)

S2 Table Characteristics of included cross-over trials. (XLSX)

S3 Table PRISMA checklist. (DOC)

S1 Text List of all included trials with references. (DOCX)

S2 Text Eggers test. (DOCX)

S1 Figs Risk of gastrointestinal adverse events in parallel group trials and cross-over trials. (DOCX)

S2 Figs Risk of gastrointestinal adverse events according to type of methylphenidate in parallel group trials. (DOCX)

S3 Figs Risk of gastrointestinal adverse events according to dose of methylphenidate in parallel group trials. (DOCX)

S4 Figs Risk of gastrointestinal adverse events according to duration of methylphenidate treatment in parallel group trials. (DOCX)

S5 Figs Effect of co-intervention on the risk of gastrointestinal adverse events in parallel group trials and cross-over trials. (DOCX)
