# Supplementary material for: Gastrointestinal adverse events during methylphenidate treatment of children and adolescents with attention deficit hyperactivity disorder: A systematic review with meta-analysis and Trial Sequential Analysis of randomised clinical trials
Source: PLoS One. 2017 Jun 15;12(6):e0178187. doi: 10.1371/journal.pone.0178187 (PMC5472278; doi:10.1371/journal.pone.0178187)
Supplement: S1 Text — (DOCX) [file pone.0178187.s004.docx]

# S1 Text

# References to articles included in ”Gastrointestinal adverse events during methylphenidate treatment of children and adolescents with attention deficit hyperactivity disorder, according to type, dose and duration: a systematic review with meta-analysis and Trial Sequential Analysis of randomized clinical trials”

**Parallel group trials**

**Biederman 2003**

Biederman J, Quinn D, Weiss M, Markabi S, Weidenman M, Edson K, et al. Efficacy and safety of Ritalin LA, a new, once daily, extended-release dosage form of methylphenidate, in children with attention deficit hyperactivity disorder. Paediatr Drugs. 2003;5(12):833–41.

**Carlson 2007**

Carlson GA, Dunn D, Kelsey D, Ruff D, Ball S, Ahrbecker L, et al. A pilot study for augmenting atomoxetine with methylphenidate: safety of concomitant therapy in children with attention-deficit/hyperactivity disorder. Child Adolesc Psychiatry Ment Health. 2007;1(1).

**Childress 2009**

*Childress AC, Spencer T, Lopez F, Gerstner O, Thulasiraman A, Muniz R, et al. Efficacy and safety of dexmethylphenidate extended-release capsules administered once daily to children with attention-deficit/hyperactivity disorder. J Child Adolesc Psychopharmacol. 2009 Aug;19(4):351–61.

Greenbaum M, Muniz R, Brams M, Boellner S, Gerstner O, Borello M, et al. Cardiovascular safety of dexmethylphenidate extended release in children with ADHD. Annals of Neurology. 2007(62):S146–S146.

Childress A, Muniz R, Miller J, Arnold V, Harper L, Gerstner O. Fixed-dose titration study of dexmethylphenidate extended release in children with ADHD: Effects on teacher-rated scales. Annals of Neurology. 2007(62):S125–S125.

Lopez F, Muniz R, Joyce J, Franklin E, Gerstner O, Borello M. Fixed-dose titration study of dexmethylphenidate extended release in children with ADHD: effects on parent-rated scales. Ann Neurol. 2007;62(Suppl 11):122.

Childress A. Dose related safety and efficacy of dexmethylphenidate ER. Brown Univ Child Adolesc Psychopharmacol Update. 2009;11(11):1–8.

**Coghill 2013**

*Coghill D, Banaschewski T, Lecendreux M, Soutullo C, Johnson M, Zuddas A, et al. European, randomized, phase 3 study of lisdexamfetamine dimesylate in children and adolescents with attention-deficit/hyperactivity disorder. Eur Neuropsychopharmacol J Eur Coll Neuropsychopharmacol. 2013 Oct;23(10):1208–18.

Zuddas A, Banaschewski T, Lecendreux M, Soutullo C, Johnson M, Anderson C, et al. Clinical efficacy of lisdexamfetamine dimesylate in children and adolescents with ADHD: A post-hoc analysis. Eur Neuropsychopharmacol J Eur Coll Neuropsychopharmacol. 2012(22):431.

Hodgkins P, Coghill D, Soutullo C, Bloomfield R, Gasior M, Johnson M. Effect of lisdexamfetamine dimesylate on functional impairment in children and adolescents with attention-deficit/hyperactivity disorder [Internet]. Abstracts presented at: the Scandinavian College of Neuropsychopharmacology (SCNP) 53rd Annual Meeting; 2012 Apr 25; Copenhagen, Denmark. Available from: Volume 24, issue supplement 1s

Gasior M, Coghill D, Soutullo C, Lyne A, Johnson M. Efficacy and safety of lisdexamfetamine dimesylate in children and adolescents with ADHD: A phase 3, randomized, double-blind, multicenter, parallel-group, placebo-and active-controlled, dose-optimized study in Europe. In Copenhagen, Denmark: John Wiley & Sons A/S; 2012.

Banaschewski T, Johnson M, Lecendreux M, Zuddas A, Adeyi B, Hodgkins P, et al. Health-related quality of life and functional outcomes from a randomized-withdrawal study of long-term lisdexamfetamine dimesylate treatment in children and adolescents with attention-deficit/hyperactivity disorder. CNS Drugs. 2014 Dec;28(12):1191–203.

Banaschewski T, Soutullo C, Lecendreux M, Johnson M, Zuddas A, Hodgkins P, et al. The child health and illness profile as a measure of health-related quality of life in stimulant-treated children and adolescents with ADHD. In Dublin, Ireland; 2013.

Coghill D, Banaschewski T, Lecendreux M, Soutullo C, Johnson M, Zuddas A, et al. The first European studies of lisdexamfetamine dimesylate in children and adolescents with attention-deficit/hyperactivity disorder. In Dublin, Ireland; 2013.

Coghill D, Banaschewski T, Lecendreux M, Soutullo C, Johnson M, Zuddas A, et al. Post hoc comparison of the efficacy of lisdexamfetamine dimesylate and osmotic-release oral system methylphenidate in children and adolescents with ADHD. In Nice, France; 2013.

Hodgkins P, Setyawan J, Banaschewski T, Soutullo C, Lecendreux M, Johnson M, et al. Health utility scores in children and adolescents with attention-deficit/hyperactivity disorder: Response to stimulant treatment. In Dublin, Ireland; 2013.

Coghill DR, Banaschewski T, Lecendreux M, Soutullo C, Zuddas A, Adeyi B, et al. Post hoc analyses of the impact of previous medication on the efficacy of lisdexamfetamine dimesylate in the treatment of attention-deficit/hyperactivity disorder in a randomized, controlled trial. Neuropsychiatr Dis Treat. 2014;10:2039–47.

Coghill DR, Banaschewski T, Lecendreux M, Zuddas A, Dittmann RW, Otero IH, et al. Efficacy of lisdexamfetamine dimesylate throughout the day in children and adolescents with attention-deficit/hyperactivity disorder: results from a randomized, controlled trial. Eur Child Adolesc Psychiatry. 2014 Feb;23(2):61–8.

Doddamani L, Hodgkins P, Adeyi B, Squires L, Civil R, Coghill DR. Functional impairment in children and adolescents with attention deficit hyperactivity disorder: Results from short-and long-term studies of lisdexamfetamine dimesylate. In Perth, WA Australia; 2014.

**Findling 2006**

Findling RL, Quinn D, Hatch SJ, Cameron SJ, DeCory HH, McDowell M. Comparison of the clinical efficacy of twice-daily Ritalin and once-daily Equasym XL with placebo in children with Attention Deficit/Hyperactivity Disorder. Eur Child Adolesc Psychiatry. 2006 Dec;15(8):450–9.

**Findling 2008**

Findling RL, Bukstein OG, Melmed RD, Lopez FA, Sallee FR, Arnold LE, et al. A randomized, double-blind, placebo-controlled, parallel-group study of methylphenidate transdermal system in pediatric patients with attention-deficit/hyperactivity disorder. J Clin Psychiatry. 2008 Jan;69(1):149–59.

Swanson JM. Transdermal methylphenidate more effective than placebo for treating ADHD. Evid Based Ment Health. 2008 Nov;11(4).

Faraone SV, Glatt SJ, Bukstein OG, Lopez FA, Arnold LE, Findling RL. Effects of once-daily oral and transdermal methylphenidate on sleep behavior of children with ADHD. J Atten Disord. 2009 Jan;12(4):308–15.

**Findling 2010**

*Findling RL, Turnbow J, Burnside J, Melmed R, Civil R, Li Y. A randomized, double-blind, multicenter, parallel-group, placebo-controlled, dose-optimization study of the methylphenidate transdermal system for the treatment of ADHD in adolescents. CNS Spectr. 2010 Jul;15(7):419–30.

Findling RL, Katic A, Rubin R, Moon E, Civil R, Li Y. A 6-month, open-label, extension study of the tolerability and effectiveness of the methylphenidate transdermal system in adolescents diagnosed with attention-deficit/hyperactivity disorder. J Child Adolesc Psychopharmacol. 2010 Oct;20(5):365–75.

Keating GM. Methylphenidate transdermal system in attention-deficit hyperactivity disorder in adolescents: profile report. Drugs RD. 2012 Sep 1;12(3):171–3.

**Greenhill 2002**

Greenhill LL, Findling RL, Swanson JM. A double-blind, placebo-controlled study of modified-release methylphenidate in children with attention-deficit/hyperactivity disorder. Pediatrics. 2002 Mar;109(3).

**Greenhill 2006**

Greenhill LL, Muniz R, Ball RR, Levine A, Pestreich L, Jiang H. Efficacy and safety of dexmethylphenidate extended-release capsules in children with attention-deficit/hyperactivity disorder. J Am Acad Child Adolesc Psychiatry. 2006 Jul;45(7):817–23.

**Lin 2014**

Lin DY, Kratochvil CJ, Xu W, Jin L, D’Souza DN, Kielbasa W, et al. A randomized trial of edivoxetine in pediatric patients with attention-deficit/hyperactivity disorder. J Child Adolesc Psychopharmacol. 2014 May;24(4).

**Newcorn 2008**

*Newcorn JH, Kratochvil CJ, Allen AJ, Casat CD, Ruff DD, Moore RJ, et al. Atomoxetine and osmotically released methylphenidate for the treatment of attention deficit hyperactivity disorder: acute comparison and differential response. Am J Psychiatry. 2008 Jun;165(6):721–30.

Toplak ME. Osmotically released methylphenidate is more effective than atomoxetine in children and adolescents with ADHD. Evid Based Ment Health. 2009 Feb;12(1).

Osmotically Released Methylphenidate Compared to Atomoxetine for ADHD. Brown Univ Child Adolesc Psychopharmacol Update. Volume 10, 2008(Issue 8):1–8.

**Palumbo 2008**

*Palumbo DR, Sallee FR, Pelham WEJ, Bukstein OG, Daviss WB, McDermott MP. Clonidine for attention-deficit/hyperactivity disorder: I. Efficacy and tolerability outcomes. J Am Acad Child Adolesc Psychiatry. 2008 Feb;47(2):180–8.

Daviss WB, Patel NC, Robb AS, McDermott MP, Bukstein OG, Pelham WEJ, et al. Clonidine for attention-deficit/hyperactivity disorder: II. ECG changes and adverse events analysis. J Am Acad Child Adolesc Psychiatry. 2008 Feb;47(2):189–98.

Cannon M, Pelham WH, Sallee FR, Palumbo DR, Bukstein O, Daviss WB. Effects of clonidine and methylphenidate on family quality of life in attention-deficit/hyperactivity disorder. J Child Adolesc Psychopharmacol. 2009 Oct;19(5):511–7.

**Pliszka 2000**

Pliszka SR, Browne RG, Olvera RL, Wynne SK. A double-blind, placebo-controlled study of Adderall and methylphenidate in the treatment of attention-deficit/hyperactivity disorder. J Am Acad Child Adolesc Psychiatry. 2000 May;39(5):619–26.

**Riggs 2011**

*Riggs PD, Winhusen T, Davies RD, Leimberger JD, Mikulich-Gilbertson S, Klein C, et al. Randomized controlled trial of osmotic-release methylphenidate with cognitive-behavioral therapy in adolescents with attention-deficit/hyperactivity disorder and substance use disorders. J Am Acad Child Adolesc Psychiatry. 2011 Sep;50(9):903–14.

Warden D, Riggs PD, Min S-J, Mikulich-Gilbertson SK, Tamm L, Trello-Rishel K, et al. Major depression and treatment response in adolescents with ADHD and substance use disorder. Drug Alcohol Depend. 2012 Jan 1;120(1-3):214–9.

Winhusen TM, Lewis DF, Riggs PD, Davies RD, Adler LA, Sonne S, et al. Subjective effects, misuse, and adverse effects of osmotic-release methylphenidate treatment in adolescent substance abusers with attention-deficit/hyperactivity disorder. J Child Adolesc Psychopharmacol. 2011 Oct;21(5):455–63.

Tamm L, Adinoff B, Nakonezny PA, Winhusen T, Riggs P. Attention-deficit/hyperactivity disorder subtypes in adolescents with comorbid substance-use disorder. Am J Drug Alcohol Abuse. 2012 Jan;38(1).

Tamm L, Trello-Rishel K, Riggs P, Nakonezny PA, Acosta M, Bailey G, et al. Predictors of treatment response in adolescents with comorbid substance use disorder and attention-deficit/hyperactivity disorder. J Subst Abuse Treat. 2013 Feb;44(2):224–30.

**Tucker 2009**

*Tucker JD, Suter W, Petibone DM, Thomas RA, Bailey NL, Zhou Y, et al. Cytogenetic assessment of methylphenidate treatment in pediatric patients treated for attention deficit hyperactivity disorder. Mutat Res. 2009 Jul;677(1-2):53–8.

Zhou Y, Muni R, Tucker JD, Kumar V. Extended-release methylphenidate exposure and the frequency of cytogenetic abnormalities in children with attention-deficit-hyperactivity disorder. Journal of child and adolescent psychopharmacology, 785. 2009.

**Wigal 2004**

Wigal S, Swanson JM, Feifel D, Sangal RB, Elia J, Casat CD, et al. A double-blind, placebo-controlled trial of dexmethylphenidate hydrochloride and d,l-threo-methylphenidate hydrochloride in children with attention-deficit/hyperactivity disorder. J Am Acad Child Adolesc Psychiatry. 2004 Nov;43(11):1406–14.

**Wilens 2006**

*Wilens TE, McBurnett K, Bukstein O, McGough J, Greenhill L, Lerner M, et al. Multisite controlled study of OROS methylphenidate in the treatment of adolescents with attention-deficit/hyperactivity disorder. Arch Pediatr Adolesc Med. 2006 Jan;160(1).

Newcorn JH, Stein MA, Cooper KM. Dose-response characteristics in adolescents with attention-deficit/hyperactivity disorder treated with OROS methylphenidate in a 4-week, open-label, dose-titration study. J Child Adolesc Psychopharmacol. 2010 Jun;20(3):187–96.

McGough JJ, McBurnett K, Bukstein O, Wilens TE, Greenhill L, Lerner M, et al. Once-daily OROS methylphenidate is safe and well tolerated in adolescents with attention-deficit/hyperactivity disorder. J Child Adolesc Psychopharmacol. 2006 Jun;16(3):351–6.

Oral system methylphenidate for teen ADHD. Brown Univ Child Adolesc Psychopharmacol. 2006(volume 8, issue 3):4–5.

Biederman J. Effectiveness and safety of the once-daily OROS formulation of methylphenidate in adolescents with attention-deficit/hyperactivity disorder. Eur Neuropsychopharmacol. 2003(Volume 13, supplement 4):S448–S448.

Greenhill L. Safety and efficacy of OROS MPH in adolescents with ADHD. In San Francisco, California; 2003

**Wolraich 2001**

*Wolraich ML, Greenhill LL, Pelham W, Swanson J, Wilens T, Palumbo D, et al. Randomized, controlled trial of oros methylphenidate once a day in children with attention-deficit/hyperactivity disorder. Pediatrics. 2001 Oct;108(4):883–92.

Wolraich ML. Efficacy and safety of OROS(r) methylphenidate HCl (mph) extended-release tablets (CONCERTA(tm)), conventional MPH, and placebo in children with ADHD. Int J Neuropsychopharmacol. 2000(3):329.

Greenhill LL. Evaluation of the efficacy and safety of Concerta (Methylphenidate HCI) extended-release tablets, ritalin, and placebo in children with ADHD. Neurology. 2000(54 (7)):A420–1.

Wolraich ML. Evaluation of efficacy and safety of OROS Methylphenidate HCI (MPH) Extended-release tablets, methylphenidate tid, and placebo in children with ADHD. Pediatr Res. 2000(47(Suppl.)):36A.

Greenhill LL. Efficacy and safety of once-daily methylphenidate HCl, standard methylphenidate and placebo in children with ADHD. In Chicago, Illinois; 2000.

Swanson JM, Greenhill LL, Pelham WH, Wilens T, Wolraich ML, Abikoff H, et al. Initiating Concerta(TM) (OROS methylphenidate HCl) qd in children with attention-deficit hyperactivity disorder. Clin Res. 2000(3(59)):76.

**Cross-over studies**

**Barkley 1989**

*Barkley RA, McMurray MB, Edelbrock CS, Robbins K. The response of aggressive and nonaggressive ADHD children to two doses of methylphenidate. J Am Acad Child Adolesc Psychiatry. 1989 Nov;(6):873–81.

Barkley RA, McMurray MB, Edelbrock CS, Robbins K. Side effects of methylphenidate in children with attention deficit hyperactivity disorder: a systemic, placebo-controlled evaluation. Pediatrics. 1990 Aug;86(2):184–92.

**Brams 2008**

Brams M, Muniz R, Childress A, Giblin J, Mao A, Turnbow J, et al. A randomized, double-blind, crossover study of once-daily dexmethylphenidate in children with attention-deficit hyperactivity disorder: rapid onset of effect. CNS Drugs. 2008 Jan;22(8):693–704.

**Brams 2012**

*Brams M, Turnbow J, Pestreich L, Giblin J, Childress A, McCague K, et al. A randomized, double-blind study of 30 versus 20 mg dexmethylphenidate extended-release in children with attention-deficit/hyperactivity disorder: late-day symptom control. J Clin Psychopharmacol. 2012 Oct;32(5):637–44.

Silva RR, Brams M, McCague K, Pestreich L, Muniz R. Extended-release dexmethylphenidate 30 mg/d versus 20 mg/d: duration of attention, behavior, and performance benefits in children with attention-deficit/hyperactivity disorder. Clin Neuropharmacol. 2013 Aug;36(4):117–21.

Dexmethylphenidate may be effective later in the day. Brown Univ Child Adolesc Psychopharmacol Update. Volume 14, Nov 2012(Issue 11):1–8.

Muniz R, Pestreich L, McCague K, Padilla A, Brams M, Childress A. Extended-Release Dexmethylphenidate 30 mg Improves Late-Day Attention Deficit Hyperactivity Disorder (ADHD) Symptom Control in Children with ADHD: A Randomized, Double-Blind Crossover Study. J Child Adolesc Psychopharmacol. December 2010(20(6)):527–38.

Padilla A, Pestreich L, McCague K, Muniz R. Late-day Attention Deficit Hyperactivity Disorder (ADHD) symptom control improvement with extended-release dexmethylphenidate in children with ADHD of all ethnicities: A sub-analysis. J Child Adolesc Psychopharmacol. December 2010(20(6)):527–38.

Brams M, Turnbow J, Pestreich L, Giblin J, Childress A, McCague K, et al. Erratum: A randomized, double-blind study of 30 versus 20 mg dexmethylphenidate extended-release in children with attention-deficit/ hyperactivity disorder: Late-day symptom control. J Clin Psychopharmacol. October 2012, volume 32(Issue 5):637–44

**Buitelaar 1995**

*Buitelaar JK, Van der Gaag RJ, Swaab-Barneveld H, Kuiper M. Prediction of clinical response to methylphenidate in children with attention-deficit hyperactivity disorder. J Am Acad Child Adolesc Psychiatry. 1995 Aug;34(8):1025–32.

Buitelaar JK, van der Gaag RJ, Swaab-Barneveld H, Kuiper M. Pindolol and methylphenidate in children with attention-deficit hyperactivity disorder. Clinical efficacy and side-effects. J Child Psychol Psychiatry. 1996 Jul;37(5):587–95.

**Bukstein 1998**

Bukstein OG, Kolko DJ. Effects of methylphenidate on aggressive urban children with attention deficit hyperactivity disorder. J Clin Child Psychol. 1998 Oct;27(3):340–51.

**Carlson 1995**

*Carlson GA, Rapport MD, Kelly KL, Pataki CS. Methylphenidate and Desipramine in Hospitalized Children with Comorbid Behavior and Mood Disorders: Separate and Combined Effects on Behavior and Mood. J Child Adolesc Psychopharmacol. 1995 Jan 1;5(3):191–204.

Pataki CS, Carlson GA, Kelly KL, Rapport MD, Biancaniello TM. Side effects of methylphenidate and desipramine alone and in combination in children. J Am Acad Child Adolesc Psychiatry. 1993 Sep;32(5):1065–72.

**Castellanos 1997**

*Castellanos FX, Giedd JN, Elia J, Marsh WL, Ritchie GF, Hamburger SD, et al. Controlled stimulant treatment of ADHD and comorbid Tourette’s syndrome: effects of stimulant and dose. J Am Acad Child Adolesc Psychiatry. 1997 May;36(5):589–96.

Castellanos FX. Stimulants and tic disorders: from dogma to data. Arch Gen Psychiatry. 1999 Apr;56(4):337–8.

**Chacko 2005**

Chacko A, Pelham WE, Gnagy EM, Greiner A, Vallano G, Bukstein O, et al. Stimulant medication effects in a summer treatment program among young children with attention-deficit/hyperactivity disorder. J Am Acad Child Adolesc Psychiatry. 2005 Mar;44(3):249–57.

**Chronis 2003**

*Chronis AM, Pelham WEJ, Gnagy EM, Roberts JE, Aronoff HR. The impact of late-afternoon stimulant dosing for children with ADHD on parent and parent-child domains. J Clin Child Adolesc Psychol Off J Soc Clin Child Adolesc Psychol Am Psychol Assoc Div 53. 2003 Mar;32(1):118–26.

Pelham WE, Gnagy EM, Chronis AM, Burrows-MacLean L, Fabiano GA, Onyango AN, et al. A comparison of morning-only and morning/late afternoon Adderall to morning-only, twice-daily, and three times-daily methylphenidate in children with attention-deficit/hyperactivity disorder. Pediatrics. 1999 Dec;104(6):1300–11.

**DuPaul 1996**

DuPaul GJ, Anastopoulos AD, Kwasnik D, Barkley RA, McMurray MB, DuPaul GJ. Methylphenidate effects on children with Attention Deficit Hyperactivity Disorder: Self-report of symptoms, side-effects, and self-esteem. J Atten Disord. 1996 Apr 1;1(1):3–15.

**Findling 2007**

Findling RL, Short EJ, McNamara NK, Demeter CA, Stansbrey RJ, Gracious BL, et al. Methylphenidate in the treatment of children and adolescents with bipolar disorder and attention-deficit/hyperactivity disorder. J Am Acad Child Adolesc Psychiatry. 2007 Nov;46(11):1445–53.

**Fine 1993**

*Fine S, Johnston C. Drug and placebo side effects in methylphenidate-placebo trial for attention deficit hyperactivity disorder. Child Psychiatry Hum Dev. 1993 Fall;24(1).

Johnston C, Fine S. Methods of evaluating methylphenidate in children with attention deficit hyperactivity disorder: acceptability, satisfaction, and compliance. J Pediatr Psychol. 1993 Dec;18(6):717–30.

**Fitzpatrick 1992**

Fitzpatrick PA, Klorman R, Brumaghim JT, Borgstedt AD. Effects of sustained-release and standard preparations of methylphenidate on attention deficit disorder. J Am Acad Child Adolesc Psychiatry. 1992 Mar;31(2):226–34.

**Klorman 1990**

*Klorman R, Brumaghim JT, Fitzpatrick PA, Borgstedt AD. Clinical effects of a controlled trial of methylphenidate on adolescents with attention deficit disorder. J Am Acad Child Adolesc Psychiatry. 1990 Sep;29(5):702–9.

Klorman R, Brumaghim JT, Fitzpatrick PA, Borgstedt AD. Methylphenidate reduces abnormalities of stimulus classification in adolescents with attention deficit disorder. J Abnorm Psychol. 1992 Feb;101(1):130–8.

Klorman R, Brumaghim JT, Fitzpatrick PA, Borgstedt AD. Methylphenidate speeds evaluation processes of attention deficit disorder adolescents during a continuous performance test. J Abnorm Child Psychol. 1991 Jun;19(3):263–83.

**Kollins 2006 (PATS)**

*Kollins S, Greenhill L, Swanson J, Wigal S, Abikoff H, McCracken J, et al. Rationale, design, and methods of the Preschool ADHD Treatment Study (PATS). J Am Acad Child Adolesc Psychiatry. 2006 Nov;45(11):1275–83.

Greenhill L, Kollins S, Abikoff H, McCracken J, Riddle M, Swanson J, et al. Efficacy and safety of immediate-release methylphenidate treatment for preschoolers with ADHD. J Am Acad Child Adolesc Psychiatry. 2006 Nov;45(11):1284–93.

Abikoff HB, Vitiello B, Riddle MA, Cunningham C, Greenhill LL, Swanson JM, et al. Methylphenidate effects on functional outcomes in the Preschoolers with Attention-Deficit/Hyperactivity Disorder Treatment Study (PATS). J Child Adolesc Psychopharmacol. 2007 Oct;17(5):581–92.

Ghuman JK, Riddle MA, Vitiello B, Greenhill LL, Chuang SZ, Wigal SB, et al. Comorbidity moderates response to methylphenidate in the Preschoolers with Attention-Deficit/Hyperactivity Disorder Treatment Study (PATS). J Child Adolesc Psychopharmacol. 2007 Oct;17(5):563–80.

March JS. The preschool ADHD Treatment Study (PATS) as the culmination of twenty years of clinical trials in pediatric psychopharmacology. J Am Acad Child Adolesc Psychiatry. 2011 May;50(5):427–30.

McGough J, McCracken J, Swanson J, Riddle M, Kollins S, Greenhill L, et al. Pharmacogenetics of methylphenidate response in preschoolers with ADHD. J Am Acad Child Adolesc Psychiatry. 2006 Nov;45(11):1314–22.

Riddle MA, Yershova K, Lazzaretto D, Paykina N, Yenokyan G, Greenhill L, et al. The Preschool Attention-Deficit/Hyperactivity Disorder Treatment Study (PATS). J Am Acad Child Adolesc Psychiatry. 2013 Mar;52(3).

Swanson J, Greenhill L, Wigal T, Kollins S, Stehli A, Davies M, et al. Stimulant-related reductions of growth rates in the PATS. J Am Acad Child Adolesc Psychiatry. 2006 Nov;45(11):1304–13.

Vitiello B, Abikoff HB, Chuang SZ, Kollins SH, McCracken JT, Riddle MA, et al. Effectiveness of methylphenidate in the 10-month continuation phase of the Preschoolers with Attention-Deficit/Hyperactivity Disorder Treatment Study (PATS). J Child Adolesc Psychopharmacol. 2007 Oct;17(5).

Wigal T, Greenhill L, Chuang S, McGough J, Vitiello B, Skrobala A, et al. Safety and tolerability of methylphenidate in preschool children with ADHD. J Am Acad Child Adolesc Psychiatry. 2006 Nov;45(11):1294–303.

Table of Contents, PATS. Brown Univ Child Adolesc Psychopharmacol Update. 2006;8(12):1–8.

PATS Shows Mixed Effect of Medication on Functional Outcomes. Brown Univ Child Adolesc Psychopharmacol Update. 2008;10(2):1–8.

Young ADHD patients may improve with low-dose meds. Psychiatr Ann. 2006(Volume 36, issue 12:826.).

Greenhill. ERRATUM “Efficacy and Safety of Immediate-Release MPH Treatment for Preschoolers With ADHD.” J Am Acad Child Adolesc Psychiatry. 46(1):141.

Kollins SH, Greenhill L. Evidence Base for the Use of Stimulant Medication in Preschool Children With ADHD. Infants Young Child [Internet]. 2006;19(2). Available from: http://journals.lww.com/iycjournal/Fulltext/2006/04000/Evidence_Base_for_the_Use_of_Stimulant_Medication.6.aspx

Riddle MA. New Findings from the Preschoolers with Attention-Deficit/Hyperactivity Disorder Treatment Study (PATS). J Child Adolesc Psychopharmacol. 2007 Oct 1;17(5):543–6.

Wagner K. Methylphenidate treatment of ADHD in preschoolers. Psychiatr Times. 2007(24(3):47.).

Greenhill L. Preschool ADHD treatment study (PATS): science and controversy. Econ Neurosci. 2001(3 (5):49–53).

Reiff M. Journal article reviews: attention-deficit/ hyperactivity disorder. J Dev Behav Pediatr. 2007;(Vol. 28, issue 1:71–2.).

**Manos 1999**

*Manos MJ, Short EJ, Findling RL. Differential effectiveness of methylphenidate and Adderall in school-age youths with attention-deficit/hyperactivity disorder. J Am Acad Child Adolesc Psychiatry. 1999 Jul;38(7):813–9.

Faraone SV, Short EJ, Biederman J, Findling RL, Roe C, Manos MJ. Efficacy of Adderall and methylphenidate in attention deficit hyperactivity disorder: a drug-placebo and drug-drug response curve analysis of a naturalistic study. Int J Neuropsychopharmacol Off Sci J Coll Int Neuropsychopharmacol CINP. 2002 Jun;5(2):121–9.

Findling RL, Short EJ, Manos MJ. Developmental aspects of psychostimulant treatment in children and adolescents with attention-deficit/hyperactivity disorder. J Am Acad Child Adolesc Psychiatry. 2001 Dec;40(12):1441–7.

Findling RL, Short EJ, Manos MJ. Short-term cardiovascular effects of methylphenidate and adderall. J Am Acad Child Adolesc Psychiatry. 2001 May;40(5):525–9.

**McBride 1988**

McBride MC. An individual double-blind crossover trial for assessing methylphenidate response in children with attention deficit disorder. J Pediatr. 1988 Jul;113(1 Pt 1):137–45.

**McGough 2006**

*McGough JJ, Wigal SB, Abikoff H, Turnbow JM, Posner K, Moon E. A randomized, double-blind, placebo-controlled, laboratory classroom assessment of methylphenidate transdermal system in children with ADHD. J Atten Disord. 2006 Feb;9(3):476–85.

Abstracts from the XXVI CINP Congress, Munich, 13–17 July 2008. Int J Neuropsychopharmacol. 2008 Jul 1;11(Supplement 1):1–338.

**Muniz 2008**

*Muniz R, Brams M, Mao A, McCague K, Pestreich L, Silva R. Efficacy and safety of extended-release dexmethylphenidate compared with d,l-methylphenidate and placebo in the treatment of children with attention-deficit/hyperactivity disorder: a 12-hour laboratory classroom study. J Child Adolesc Psychopharmacol. 2008 Jun;18(3):248–56.

Silva R, Muniz R, McCague K, Childress A, Brams M, Mao A. Treatment of children with attention-deficit/hyperactivity disorder: results of a randomized, multicenter, double-blind, crossover study of extended-release dexmethylphenidate and D,L-methylphenidate and placebo in a laboratory classroom setting. Psychopharmacol Bull. 2008;41(1).

**Murray 2011**

Murray DW, Childress A, Giblin J, Williamson D, Armstrong R, Starr HL. Effects of OROS methylphenidate on academic, behavioral, and cognitive tasks in children 9 to 12 years of age with attention-deficit/hyperactivity disorder. Clin Pediatr (Phila). 2011 Apr;50(4):308–20.

**Musten 1997**

*Musten LM, Firestone P, Pisterman S, Bennett S, Mercer J. Effects of methylphenidate on preschool children with ADHD: cognitive and behavioral functions. J Am Acad Child Adolesc Psychiatry. 1997 Oct;36(10):1407–15.

Firestone P, Musten LM, Pisterman S, Mercer J, Bennett S. Short-term side effects of stimulant medication are increased in preschool children with attention-deficit/hyperactivity disorder: a double-blind placebo-controlled study. J Child Adolesc Psychopharmacol. 1998;8(1).

**Pearson 2013**

*Pearson DA, Santos CW, Aman MG, Arnold LE, Casat CD, Mansour R, et al. Effects of extended release methylphenidate treatment on ratings of attention-deficit/hyperactivity disorder (ADHD) and associated behavior in children with autism spectrum disorders and ADHD symptoms. J Child Adolesc Psychopharmacol. 2013 Jun;23(5):337–51.

Methylphenidate dosing improved behavior in children with ASD. Brown Univ Child Adolesc Psychopharmacol Update. AUG 2013, volume 15(Issue 8):1–8.

**Pelham 1990**

Pelham WEJ, Greenslade KE, Vodde-Hamilton M, Murphy DA, Greenstein JJ, Gnagy EM, et al. Relative efficacy of long-acting stimulants on children with attention deficit-hyperactivity disorder: a comparison of standard methylphenidate, sustained-release methylphenidate, sustained-release dextroamphetamine, and pemoline. Pediatrics. 1990 Aug;86(2):226–37.

**Pelham 1999**

Pelham WE, Aronoff HR, Midlam JK, Shapiro CJ, Gnagy EM, Chronis AM, et al. A comparison of ritalin and adderall: efficacy and time-course in children with attention-deficit/hyperactivity disorder. Pediatrics. 1999 Apr;103(4).

**Pelham 2001**

Pelham WE, Gnagy EM, Burrows-Maclean L, Williams A, Fabiano GA, Morrisey SM, et al. Once-a-day Concerta methylphenidate versus three-times-daily methylphenidate in laboratory and natural settings. Pediatrics. 2001 Jun;107(6).

**Pelham 2005**

*Pelham WEJ, Manos MJ, Ezzell CE, Tresco KE, Gnagy EM, Hoffman MT, et al. A dose-ranging study of a methylphenidate transdermal system in children with ADHD. J Am Acad Child Adolesc Psychiatry. 2005 Jun;44(6):522–9.

Pelham WE, Burrows-Maclean L, Gnagy EM, Fabiano GA, Coles EK, Tresco KE, et al. Transdermal methylphenidate, behavioral, and combined treatment for children with ADHD. Exp Clin Psychopharmacol. 2005 May;13(2):111–26.

Chacko A, Pelham WE, Gnagy EM, Greiner A, Vallano G, Bukstein O, et al. Stimulant medication effects in a summer treatment program among young children with attention-deficit/hyperactivity disorder. J Am Acad Child Adolesc Psychiatry. 2005 Mar;44(3):249–57.

**Pelham 2011**

Pelham WE, Waxmonsky JG, Schentag J, Ballow CH, Panahon CJ, Gnagy EM, et al. Efficacy of a methylphenidate transdermal system versus t.i.d. methylphenidate in a laboratory setting. J Atten Disord. 2011 Jan;15(1).

**Quinn 2004**

Quinn D, Wigal S, Swanson J, Hirsch S, Ottolini Y, Dariani M, et al. Comparative pharmacodynamics and plasma concentrations of d-threo-methylphenidate hydrochloride after single doses of d-threo-methylphenidate hydrochloride and d,l-threo-methylphenidate hydrochloride in a double-blind, placebo-controlled, crossover laboratory school study in children with attention-deficit/hyperactivity disorder. J Am Acad Child Adolesc Psychiatry. 2004 Nov;43(11):1422–9.

**Ramtvedt 2013**

*Ramtvedt BE, Roinas E, Aabech HS, Sundet KS. Clinical gains from including both dextroamphetamine and methylphenidate in stimulant trials. J Child Adolesc Psychopharmacol. 2013 Nov;23(9).

Ramtvedt BE, Sundet K. Relationships between computer-based testing and behavioral ratings in the assessment of attention and activity in a pediatric ADHD stimulant crossover trial. Clin Neuropsychol. 2014;28(7):1146–61.

Ramtvedt BE, Aabech HS, Sundet K. Minimizing adverse events while maintaining clinical improvement in a pediatric attention-deficit/hyperactivity disorder crossover trial with dextroamphetamine and methylphenidate. J Child Adolesc Psychopharmacol. 2014 Apr;24(3):130–9.

Ramtvedt BE, Sandvik L, Sundet K. Correspondence between children’s and adults’ ratings of stimulant-induced changes in ADHD behaviours in a crossover trial with medication-naive children. Eur J Dev Psychol. 2014 Jun 17;11(6):687–700.

**Rapport 2008**

Rapport MD, Kofler MJ, Coiro MM, Raiker JS, Sarver DE, Alderson RM. Unexpected effects of methylphenidate in attention-deficit/hyperactivity disorder reflect decreases in core/secondary symptoms and physical complaints common to all children. J Child Adolesc Psychopharmacol. 2008 Jun;18(3):237–47.

**Schulz 2010**

Schulz E, Fleischhaker C, Hennighausen K, Heiser P, Haessler F, Linder M, et al. A randomized, rater-blinded, crossover study comparing the clinical efficacy of Ritalin((R)) LA (methylphenidate) treatment in children with attention-deficit hyperactivity disorder under different breakfast conditions over 2 weeks. Atten Deficit Hyperact Disord. 2010 Nov;2(3):133–8.

**Sharp 1999**

*Sharp WS, Walter JM, Marsh WL, Ritchie GF, Hamburger SD, Castellanos FX. ADHD in girls: clinical comparability of a research sample. J Am Acad Child Adolesc Psychiatry. 1999 Jan;38(1):40–7.

Schmidt ME, Kruesi MJ, Elia J, Borcherding BG, Elin RJ, Hosseini JM, et al. Effect of dextroamphetamine and methylphenidate on calcium and magnesium concentration in hyperactive boys. Psychiatry Res. 1994 Nov;54(2).

Elia J, Welsh PA, Gullotta CS, Rapoport JL. Classroom academic performance: improvement with both methylphenidate and dextroamphetamine in ADHD boys. J Child Psychol Psychiatry. 1993 Jul;34(5).

Elia J, Borcherding BG, Rapoport JL, Keysor CS. Methylphenidate and dextroamphetamine treatments of hyperactivity: are there true nonresponders? Psychiatry Res. 1991 Feb;36(2):141–55.

Castellanos FX, Elia J, Kruesi MJ, Marsh WL, Gulotta CS, Potter WZ, et al. Cerebrospinal fluid homovanillic acid predicts behavioral response to stimulants in 45 boys with attention deficit/hyperactivity disorder. Neuropsychopharmacol Off Publ Am Coll Neuropsychopharmacol. 1996 Feb;14(2):125–37.

Borcherding BG, Keysor CS, Cooper TB, Rapoport JL. Differential effects of methylphenidate and dextroamphetamine on the motor activity level of hyperactive children. Neuropsychopharmacol Off Publ Am Coll Neuropsychopharmacol. 1989 Dec;2(4):255–63.

**Silva 2006**

Silva RR, Muniz R, Pestreich L, Childress A, Brams M, Lopez FA, et al. Efficacy and duration of effect of extended-release dexmethylphenidate versus placebo in schoolchildren with attention-deficit/hyperactivity disorder. J Child Adolesc Psychopharmacol. 2006 Jun;16(3):239–51.

**Silva 2008**

*Silva RR, Muniz R, Pestreich L, Brams M, Mao AR, Childress A, et al. Dexmethylphenidate extended-release capsules in children with attention-deficit/hyperactivity disorder. J Am Acad Child Adolesc Psychiatry. 2008 Feb;47(2).

Silva RR, Muniz R, Pestreich L, Lopez F, Childress A, Wang J. Once-daily dexmethylphenidate: A placebo-controlled crossover study in children with attention-deficit/hyperacti

**Smith 1998**

*Smith BH, Pelham WE, Evans S, Gnagy E, Molina B, Bukstein O, et al. Dosage effects of methylphenidate on the social behavior of adolescents diagnosed with attention-deficit hyperactivity disorder. Exp Clin Psychopharmacol. 1998 May;6(2).

Smith BH, Pelham WEJ, Gnagy E, Molina B, Evans S. The reliability, validity, and unique contributions of self-report by adolescents receiving treatment for attention-deficit/hyperactivity disorder. J Consult Clin Psychol. 2000 Jun;68(3):489–99.

Evans SW, Pelham WE, Smith BH, Bukstein O, Gnagy EM, Greiner AR, et al. Dose-response effects of methylphenidate on ecologically valid measures of academic performance and classroom behavior in adolescents with ADHD. Exp Clin Psychopharmacol. 2001 May;9(2):163–75.

Smith BH, Pelham WE, Gnagy E, Yudell RS. Equivalent effects of stimulant treatment for attention-deficit hyperactivity disorder during childhood and adolescence. J Am Acad Child Adolesc Psychiatry. 1998 Mar;37(3):314–21.

**Stein 1996**

Stein MA, Blondis TA, Schnitzler ER, O’Brien T, Fishkin J, Blackwell B, et al. Methylphenidate dosing: twice daily versus three times daily. Pediatrics. 1996 Oct;98(4 Pt 1):748–56.

**Stein 2003**

*Stein MA, Sarampote CS, Waldman ID, Robb AS, Conlon C, Pearl PL, et al. A dose-response study of OROS methylphenidate in children with attention-deficit/hyperactivity disorder. Pediatrics. 2003 Nov;112(5).

Stein MA, Waldman ID, Sarampote CS, Seymour KE, Robb AS, Conlon C, et al. Dopamine transporter genotype and methylphenidate dose response in children with ADHD. Neuropsychopharmacol Off Publ Am Coll Neuropsychopharmacol. 2005 Jul;30(7):1374–82.

Stein M, Sarampote CS, Seymour KE. Insomnia and tiredness in ADHD youth: relationship with methylphenidate dose, age, and weight. In San Francisco, CA. Baltimore; 2004.

Stein MA, Seymour KE, Black D, Sarampote CS, Robb A, Conlon C. Effects and side effects of Concerta methylphenidate (MPH) in children with ADHD and comorbid internalizing symptoms. In Seattle, Washington. Baltimore: International Pediatric Research Foundation; 2003.

**Stein 2011**

*Stein MA, Waldman ID, Charney E, Aryal S, Sable C, Gruber R, et al. Dose effects and comparative effectiveness of extended release dexmethylphenidate and mixed amphetamine salts. J Child Adolesc Psychopharmacol. 2011 Dec;21(6):581–8.

Wiebe S, Gruber R, Charney E, Aryal S, Waldman I, Newcorn J, et al. Sleep and emotional reactivity to extended release dexmethylphenidate versus mixed amphetamine salts: A double-blind, placebo controlled study. In Amsterdam, Netherlands; 2010. p. 82.

**Swanson 2004**

*Swanson JM, Wigal SB, Wigal T, Sonuga-Barke E, Greenhill LL, Biederman J, et al. A comparison of once-daily extended-release methylphenidate formulations in children with attention-deficit/hyperactivity disorder in the laboratory school (the Comacs Study). Pediatrics. 2004 Mar;113(3 Pt 1):e206–16.

Sonuga-Barke EJS, Swanson JM, Coghill D, DeCory HH, Hatch SJ. Efficacy of two once-daily methylphenidate formulations compared across dose levels at different times of the day: preliminary indications from a secondary analysis of the COMACS study data. BMC Psychiatry. 2004;4.

Sonuga-Barke EJS, Coghill D, Markowitz JS, Swanson JM, Vandenberghe M, Hatch SJ. Sex differences in the response of children with ADHD to once-daily formulations of methylphenidate. J Am Acad Child Adolesc Psychiatry. 2007 Jun;46(6):701–10.

Sonuga-Barke EJS, Van Lier P, Swanson JM, Coghill D, Wigal S, Vandenberghe M, et al. Heterogeneity in the pharmacodynamics of two long-acting methylphenidate formulations for children with attention deficit/hyperactivity disorder. A growth mixture modelling analysis. Eur Child Adolesc Psychiatry. 2008 Jun;17(4):245–54.

Sonuga-Barke EJS, Coghill D, Wigal T, DeBacker M, Swanson J. Adverse reactions to methylphenidate treatment for attention-deficit/hyperactivity disorder: structure and associations with clinical characteristics and symptom control. J Child Adolesc Psychopharmacol. 2009 Dec;19(6):683–90.

**Wigal 2014**

Wigal SB, Greenhill LL, Nordbrock E, Connor DF, Kollins SH, Adjei A, et al. A randomized placebo-controlled double-blind study evaluating the time course of response to methylphenidate hydrochloride extended-release capsules in children with attention-deficit/hyperactivity disorder. J Child Adolesc Psychopharmacol. 2014 Dec;24(10):562–9

**Wilens 2010**

*Wilens TE, Hammerness P, Martelon M, Brodziak K, Utzinger L, Wong P. A controlled trial of the methylphenidate transdermal system on before-school functioning in children with attention-deficit/hyperactivity disorder. J Clin Psychiatry. 2010 May;71(5):548–56.

Wilens TE, Hammerness P, Utzinger L, Georgiopoulos A, Doyle A, Brodziak K, et al. Before-School ADHD Symptoms and Functioning in Youth Treated with the Methylphenidate Transdermal Patch (MTS). J Child Adolesc Psychopharmacol.

**Zeni 2009**

Zeni CP, Tramontina S, Ketzer CR, Pheula GF, Rohde LA. Methylphenidate combined with aripiprazole in children and adolescents with bipolar disorder and attention-deficit/hyperactivity disorder: a randomized crossover trial. J Child Adolesc Psychopharmacol. 2009 Oct;19(5):553–61.

* primary reference for this study
