# Supplementary material for: Gastrointestinal adverse events during methylphenidate treatment of children and adolescents with attention deficit hyperactivity disorder: A systematic review with meta-analysis and Trial Sequential Analysis of randomised clinical trials
Source: PLoS One. 2017 Jun 15;12(6):e0178187. doi: 10.1371/journal.pone.0178187 (PMC5472278; doi:10.1371/journal.pone.0178187)
Supplement: S2 Text — (DOCX) [file pone.0178187.s005.docx]

S2 Text

Eggers test for decreased appetite

. metan events1 noevent1 events2 noevent2,label(namevar= name) rr randomi

Study | RR [95% Conf. Interval] % Weight

---------------------+---------------------------------------------------

Biederman 2003 | 5.455 0.267 111.540 1.33

Childress 2009 | 2.538 0.786 8.193 6.93

Coghill 2013 | 5.616 1.694 18.621 6.70

Findling 2006 | 2.455 0.129 46.652 1.39

Findling 2008 | 3.970 1.392 11.325 8.14

Findling 2010 | 18.372 2.572 131.226 2.94

Greenhill 2002 | 2.298 1.624 3.251 21.79

Greenhill 2006 | 3.547 1.275 9.865 8.43

Lin 2014 | 12.278 3.841 39.248 7.01

Newcorn 2008 | 6.251 1.544 25.305 5.26

Pliszka 2000 | 6.333 0.349 114.813 1.44

Riggs 2011 | 2.796 1.350 5.790 12.81

Tucker 2009 | 43.278 2.683 698.019 1.55

Wigal 2004 | 8.978 0.535 150.712 1.51

Wilens 2006 | 5.170 0.252 106.182 1.33

Wolraich 2001 | 1.581 0.706 3.539 11.44

---------------------+---------------------------------------------------

D+L pooled RR | 3.660 2.562 5.228 100.00

---------------------+---------------------------------------------------

Heterogeneity chi-squared = 20.88 (d.f. = 15) p = 0.141

I-squared (variation in RR attributable to heterogeneity) = 28.2%

Estimate of between-study variance Tau-squared = 0.1206

Test of RR=1 : z= 7.13 p = 0.000

. metabias _ES _selogES

Note: default data input format (theta, se_theta) assumed.

Tests for Publication Bias

Begg's Test

adj. Kendall's Score (P-Q) = 42

Std. Dev. of Score = 22.21

Number of Studies = 16

z = 1.89

Pr > |z| = 0.059

z = 1.85 (continuity corrected)

Pr > |z| = 0.065 (continuity corrected)

Egger's test

------------------------------------------------------------------------------

Std_Eff | Coef. Std. Err. t P>|t| [95% Conf. Interval]

-------------+----------------------------------------------------------------

slope | .3794354 1.6374 0.23 0.820 -3.132439 3.89131

bias | 8.717674 3.331681 2.62 0.020 1.571928 15.86342

------------------------------------------------------------------------------

.

 Egger’s test is performed to access the presence of publication bias. In this regression equation where the dependent variable is the standardised effect size and the independed variables is the precision of the effect size. If the intercept (bias) value is too far from zero. Thus, indicates a potentials source of publication bias.

Egger’s regression intercept (bias) is 8.717 (two-tailed p-value = 0.020). There is a publication bias in this meta-analysis.

Eggers test for nausea

metan e1 t1 e2 t2, label(namevar=studyid) random rr

Study | RR [95% Conf. Interval] % Weight

---------------------+---------------------------------------------------

Carlson 2007 | 0.333 0.015 7.240 2.06

Childress 2009 | 0.708 0.251 1.999 15.62

Coghill 2013 | 2.532 0.689 9.307 10.52

Findling 2008 | 3.107 0.663 14.562 7.70

Findling 2010 | 3.258 0.760 13.968 8.59

Greenhill 2006 | 1.695 0.447 6.432 10.07

Newcorn 2008 | 0.747 0.294 1.900 18.60

Riggs 2011 | 0.900 0.356 2.274 18.82

Wigal 2004 | 4.300 0.568 32.555 4.64

Wilens 2006 | 0.523 0.048 5.663 3.39

---------------------+---------------------------------------------------

D+L pooled RR | 1.265 0.809 1.976 100.00

---------------------+---------------------------------------------------

Heterogeneity chi-squared = 10.00 (d.f. = 9) p = 0.351

I-squared (variation in RR attributable to heterogeneity) = 10.0%

Estimate of between-study variance Tau-squared = 0.0519

Test of RR=1 : z= 1.03 p = 0.303

. metabias _ES _selogES

Note: default data input format (theta, se_theta) assumed.

Tests for Publication Bias

Begg's Test

adj. Kendall's Score (P-Q) = 17

Std. Dev. of Score = 11.18

Number of Studies = 10

z = 1.52

Pr > |z| = 0.128

z = 1.43 (continuity corrected)

Pr > |z| = 0.152 (continuity corrected)

Egger's test

------------------------------------------------------------------------------

Std_Eff | Coef. Std. Err. t P>|t| [95% Conf. Interval]

-------------+----------------------------------------------------------------

slope | .1823387 1.077588 0.17 0.870 -2.302583 2.667261

bias | 2.167359 1.609938 1.35 0.215 -1.545164 5.879883

Egger’s test is performed to access the presence of publication bias. In this regression equation where the dependent variable is the standardised effect size and the independed variables is the precision of the effect size. If the intercept (bias) value is too far from zero. Thus, indicates a potentials source of publication bias.

Egger’s regression intercept (bias) is 2.16 (two-tailed p-value = 0.215). Thus, there is no statistical significance to conclude there is a publication bias in this meta-analysis.

Eggers test for abdominal pain

metan e1 t1 e2 t2, label(namevar=studyid) random rr

Study | RR [95% Conf. Interval] % Weight

---------------------+---------------------------------------------------

Carlson 2007 | 0.333 0.015 7.240 0.81

Childress 2009 | 1.122 0.503 2.500 11.94

Coghill 2013 | 0.672 0.195 2.320 5.00

Findling 2006 | 1.035 0.292 3.670 4.79

Greenhill 2002 | 1.402 0.878 2.241 34.93

Greenhill 2006 | 5.895 0.312 111.304 0.89

Newcorn 2008 | 1.780 0.633 5.008 7.17

Pliszka 2000 | 2.591 0.112 59.932 0.78

Riggs 2011 | 0.972 0.585 1.616 29.73

Wigal 2004 | 1.830 0.211 15.888 1.64

Wilens 2006 | 0.523 0.048 5.663 1.35

Wolraich 2001 | 5.772 0.345 96.700 0.97

---------------------+---------------------------------------------------

D+L pooled RR | 1.196 0.907 1.578 100.00

---------------------+---------------------------------------------------

Heterogeneity chi-squared = 6.45 (d.f. = 11) p = 0.842

I-squared (variation in RR attributable to heterogeneity) = 0.0%

Estimate of between-study variance Tau-squared = 0.0000

Test of RR=1 : z= 1.27 p = 0.205

. metabias _ES _selogES

Note: default data input format (theta, se_theta) assumed.

Tests for Publication Bias

Begg's Test

adj. Kendall's Score (P-Q) = 16

Std. Dev. of Score = 14.58

Number of Studies = 12

z = 1.10

Pr > |z| = 0.273

z = 1.03 (continuity corrected)

Pr > |z| = 0.304 (continuity corrected)

Egger's test

------------------------------------------------------------------------------

Std_Eff | Coef. Std. Err. t P>|t| [95% Conf. Interval]

-------------+----------------------------------------------------------------

slope | .8487142 .337101 2.52 0.031 .0976064 1.599822

bias | 1.117149 .6886516 1.62 0.136 -.4172624 2.65156

------------------------------------------------------------------------------

Egger’s test is performed to access the presence of publication bias. In this regression equation where the dependent variable is the standardised effect size and the independed variables is the precision of the effect size. If the intercept (bias) value is too far from zero. Thus, indicates a potentials source of publication bias.

Egger’s regression intercept (bias) is 1.11(two-tailed p-value = 0.136). Thus, there is no statistical significance to conclude there is a publication bias in this meta-analysis.

Eggers test for vomiting

metan e1 t1 e2 t2, label(namevar=studyid) random rr

Study | RR [95% Conf. Interval] % Weight

---------------------+---------------------------------------------------

Biederman 2003 | 0.224 0.011 4.587 2.09

Carlson 2007 | 0.900 0.065 12.380 2.76

Childress 2009 | 7.588 0.453 126.952 2.39

Coghill 2013 | 3.861 0.438 34.009 4.01

Findling 2006 | 0.701 0.133 3.705 6.85

Findling 2008 | 2.003 0.639 6.278 14.55

Greenhill 2006 | 0.891 0.130 6.088 5.14

Newcorn 2008 | 0.687 0.213 2.219 13.82

Riggs 2011 | 1.136 0.573 2.252 40.57

Wigal 2004 | 1.375 0.289 6.543 7.81

---------------------+---------------------------------------------------

D+L pooled RR | 1.178 0.762 1.822 100.00

---------------------+---------------------------------------------------

Heterogeneity chi-squared = 6.29 (d.f. = 9) p = 0.711

I-squared (variation in RR attributable to heterogeneity) = 0.0%

Estimate of between-study variance Tau-squared = 0.0000

Test of RR=1 : z= 0.74 p = 0.461

. metabias _ES _selogES

Note: default data input format (theta, se_theta) assumed.

Tests for Publication Bias

Begg's Test

adj. Kendall's Score (P-Q) = 13

Std. Dev. of Score = 11.18

Number of Studies = 10

z = 1.16

Pr > |z| = 0.245

z = 1.07 (continuity corrected)

Pr > |z| = 0.283 (continuity corrected)

Egger's test

------------------------------------------------------------------------------

Std_Eff | Coef. Std. Err. t P>|t| [95% Conf. Interval]

-------------+----------------------------------------------------------------

slope | .5118804 .8555537 0.60 0.566 -1.46103 2.484791

bias | 1.437354 1.216565 1.18 0.271 -1.368049 4.242757

------------------------------------------------------------------------------

Egger’s test is performed to access the presence of publication bias. In this regression equation where the dependent variable is the standardised effect size and the independed variables is the precision of the effect size. If the intercept (bias) value is too far from zero. Thus, indicates a potentials source of publication bias.

Egger’s regression intercept (bias) is 1.438 (two-tailed p-value = 0.271). Thus, there is no statistical significance to conclude there is a publication bias in this meta-analysis.
