# Supplementary material for: Gastrointestinal adverse events during methylphenidate treatment of children and adolescents with attention deficit hyperactivity disorder: A systematic review with meta-analysis and Trial Sequential Analysis of randomised clinical trials
Source: PLoS One. 2017 Jun 15;12(6):e0178187. doi: 10.1371/journal.pone.0178187 (PMC5472278; doi:10.1371/journal.pone.0178187)
Supplement: S1 Figs — (DOCX) [file pone.0178187.s006.docx]

S1 Figs

Gastrointestinal adverse effects of methylphenidate compared with placebo or no intervention in parallel group trials and cross-over trials

Abdominal pain

Diarrhea

Dyspepsia

Increased appetite

Nausea

Vomiting

Caption: IV: inverse variance, Random: random-effect model. CI: confidence interval.
