# Supplementary material for: Gastrointestinal adverse events during methylphenidate treatment of children and adolescents with attention deficit hyperactivity disorder: A systematic review with meta-analysis and Trial Sequential Analysis of randomised clinical trials
Source: PLoS One. 2017 Jun 15;12(6):e0178187. doi: 10.1371/journal.pone.0178187 (PMC5472278; doi:10.1371/journal.pone.0178187)
Supplement: S5 Figs — (DOCX) [file pone.0178187.s010.docx]

**S5 Figs**

**Effect of co-intervention on the risk of gastrointestinal adverse events in parallel group trials and cross-over trials**

Parallel group trials: Abdominal pain

Parallel group trials: Decreased weight

Parallel group trials: Dyspepsia

Parallel group trials: Nausea

Parallel group trials: Vomiting

Cross-over trials: Abdominal pain

Cross-over trials: Decreased appetite

Cross-over trials: Nausea
